# Surgical Treatment and Outcome of Acquired Midline Palate Defects in Cats

**DOI:** 10.3389/fvets.2022.922047

**Published:** 2022-07-04

**Authors:** Ana C. Castejón-González, Darko Stefanovski, Alexander M. Reiter

**Affiliations:** ^1^Department of Clinical Sciences and Advanced Medicine, School of Veterinary Medicine, University of Pennsylvania, Philadelphia, PA, United States; ^2^Department of Clinical Studies-New Bolton Center, School of Veterinary Medicine, University of Pennsylvania, Philadelphia, PA, United States

**Keywords:** acquired palate defect, feline, palate repair, midface trauma, high-rise syndrome, maxillofacial trauma

## Abstract

Acquired midline palate defects (PDE) affect the hard palate and/or soft palate, and result from trauma, commonly falling from a height or being hit by a motor vehicle. Additional life-threating injuries and costs associated with the treatment may delay the surgical treatment. This retrospective study describes signalment, cause, and extent of the PDE, and treatment in 25 cats. In addition, the outcome of the surgical repair is described in 19 (76%) cats. All defects were repaired within 5 days of the injury. Twenty (80%) cats were 4 years of age or younger. The most common rostral extent of the PDE was to the level of the third premolar tooth (*n* = 8; 32%), incisor teeth (*n* = 7; 28%), and fourth premolar tooth (*n* = 5; 20%). The soft palate laceration was present in all cases. Surgical therapy was successful in all cases with follow-up. The most common techniques used for the closure of the hard palate defect were bilateral pedicle flaps with lateral releasing incisions (*n* = 8; 32%), direct apposition of the oral mucosa (*n* = 7; 28%), bilateral pedicle flaps with lateral releasing incisions and interquadrant splinting (*n* = 5; 20%), and unilateral pedicle flap with one lateral releasing incision (*n* = 4; 16%). A tension-free closure by direct apposition of the edges was possible for the soft palate laceration. No oronasal fistulae were identified at follow-up. The only complication was malocclusion. The interquadrant splinting was most often used for PDE extending to the rostral portion of the hard palate (*p* < 0.05). The cats that suffered postoperative malocclusion were significantly more likely to have sustained temporomandibular joint injury, underwent CT scan, or had a feeding tube placed before discharge. The results of this retrospective study indicate that the early treatment (within 5 days) of the acquired longitudinal defects in the midline of the hard and soft palates is highly successful.

## Introduction

The acquired midline palate defects (PDE) result from trauma and are frequently diagnosed after falling from a height or road traffic accidents (1–3). The hard palate is formed by the paired incisive, maxillary, and palatine bones that are fused together at the intermaxillary suture. The separation of the median palatine suture or fracture of the bones of the hard palate occurs frequently with multiple and bilateral fractures of the midface (incisive bone, maxilla and palatine bone, orbit, zygomatic arch, and nasopharynx) (2, 4). Dental, ocular, and neurologic injuries also are common. The two recent studies describe the distribution of fractures in cats with head trauma, with about 45% of the cats showing injury of the median palatine suture or bones of the hard palate (2, 3). No further description was made regarding the presence of multiple fractures, bone loss, or whether a soft tissue defect; therefore, oronasal communication was present. A study investigating cats with a history of high-rise syndrome reported 20.5% of cats having fracture of the bones of the hard palate with or without torn soft palate tissues, with the percentage increasing to 78.6% in the group evaluated by a veterinary dentist as compared to emergency and critical care clinician (8.7%) (1).

Epistaxis and oral bleeding with or without difficulty breathing, pain, facial asymmetry, and malocclusion can be associated with acute midline PDE. When such defects occur after a traumatic event, their assessment and repair may be delayed in favor of first addressing other life-threatening injuries (1, 5). The need for repair also may be underestimated due the false assumption that PDE heal on their own. Small palate defects may indeed heal by second intention. However, if defects of the palatal mucoperiosteum with involvement of the underlying bone do not fully heal, patients may have significantly decreased quality of life due to oronasal fistula development, chronic rhinitis, and possible aspiration pneumonia (5, 6).

Conservative management of a midline PDE has been historically recommended for narrow linear defects (7). Other authors recommended surgical repair as soon as the patient is clinically stable, regardless of the defect dimensions (1, 8). Surgical treatment includes apposition of the medial edges of the mucoperiosteum with or without lateral releasing incisions and elevation of mucoperiosteal flaps. Reduction and stabilization of bone fragments with a K-wire and a tension band through the upper jaw or with a wire-reinforced interquadrant bis-acryl composite splint have been described (1, 3). Still, there are no clear guidelines about the need or timing of the repair, the preferred surgical technique, or when further stabilization of the injured palate is warranted (1–3, 8).

The main objective of this study was to describe the presentation, location, extent, treatment, outcome, and complications of acquired midline PDE in client-owned cats. A second objective was to make specific recommendations for the surgical management of such PDE.

## Materials and Methods

Medical records of client-owned cats with maxillofacial trauma presented to the dentistry and oral surgery service of a specialty teaching hospital from 2000 to 2021 were reviewed. Cats were included in this study if an acquired midline PDE causing oronasal communication was diagnosed by means of an oral examination and surgical repair was attempted. Patients were excluded if the oronasal communication was not located in the midline of the palate, the defect affected only the incisive bones (between the incisor teeth and palatine fissures), or the patient presented with an oronasal fistula (edges of the PDE were epithelialized). Information about signalment and body weight of the cats, cause and extent of the PDE, diagnostic imaging findings by means of dental radiography and/or computed tomography (CT), treatment, and outcome (healing and complications) were retrieved from the medical records.

The extent of the PDE was classified from the most rostrally affected aspect of the hard palate to the transition of the hard and soft palate (THSP) or to the soft palate (if it extended more than a few mm in the soft palate). The most rostral location was leveled with the teeth. The categorization of the defects according to the width (in mm) was attempted, but such data were only available for a few cats; therefore, this information was not assessed. The anatomical functional regions that were fractured were identified in patients with CT according to the classification described in a previous study (2).

The time from trauma to surgical repair was assessed. Treatment was categorized as (a) direct medial mucoperiosteal apposition, (b) medial mucoperiosteal apposition with only one pedicle flap (unilateral releasing incision 1–2 mm away from the gingival sulcus of the maxillary teeth), (c) medial mucoperiosteal apposition with bilateral pedicle flaps (bilateral releasing incisions 1–2 mm away from the gingival sulcus of the maxillary teeth), and (d) combinations of these techniques with the addition of the interquadrant splinting to reduce and stabilize the median palatine suture separation. The mucoperiosteum between the defect and the lateral releasing incisions was elevated as deemed necessary by the clinician; thus, allowing a tension-free closure in the midline of the palate. All techniques included a gentle debridement of the soft tissue edges prior to the closure and flushing of the nasal cavity to remove blood clots and debris before and after the PDE repair. The closure of the PDE was classified as one-layer (full-thickness sutures in the oral mucosa), two-layer (deep sutures in the muscular layer [soft palate] or connective tissue of the mucoperiosteum [hard palate] and a superficial layer of sutures in the oral mucosa) or a three-layer closure (deep sutures in nasal mucosa, intermediate sutures in the muscular layer, and superficial sutures in the oral mucosa). The suture patterns and suture materials were also noted. The lateral releasing incisions had been left to heal by second intention.

The outcome was defined as good at the follow-up visits if there was no evidence of oronasal fistula after the healing period upon oral examination of the conscious patient. The evaluation was performed under anesthesia only if the patient needed further treatment (e.g., tooth extraction, splint removal, etc.). Healing was defined as continuity of the oral mucosa with no evidence of linear inflammation (redness). The inflammation attributed to the suture material in the absence of a midline defect was considered as “healed” at the time of the follow-up evaluation. The lateral defects were considered healed when the bed of granulation tissue was completely covered by epithelium, with no exposed bone. For each cat, only one healing time was recorded, corresponding to the time of healing of the PDE and any lateral releasing incisions made at the time of the repair. Information about the potential postoperative complications (oronasal fistula and malocclusion) also was retrieved from the medical records.

All analyses were conducted with Stata 17MP, StataCorp, College Station TX, with two-sided tests of hypotheses and a *p* < 0.05 as the criterion for statistical significance. The descriptive analyses included the computation of medians and range. The categorical variables were reported as frequency counts and percentages. The Spearman rank correlation was conducted to identify significant association between the independent variables with the extent of the PDE and the complications as outcomes considered. In addition, the analysis was conducted to identify whether the use of a CT was correlated with any independent variables. The independent variables studied were gender, breed, age, weight, cause of trauma, time from trauma to repair in days, presence of soft tissue trauma in the head, concurrent temporomandibular (TMJ) injury, mandibular fracture (TMJ excluded), symphyseal separation, orbital or ocular injury (soft tissue injuries and/or fractures of the orbital bones), presence of head injury (discussed further in the next paragraph), lesions of other body parts excluding the head, treatment performed, placement of a feeding tube, and diagnostic imaging by means of a CT. To further explore the association between the extent of the PDE and treatment, the variable treatment was split into the following two new variables: Use of interquadrant splinting between the maxillary canine teeth and the type of soft tissue closure (direct apposition, unilateral pedicle flap, or bilateral pedicle flaps).

For the statistical purposes, head injury was categorized as (1) no injury (beyond obvious injury of the palate), (2) fracture of the mandible (excluding TMJ), (3) ocular or orbital injury, (4) concurrent fracture of the mandible and injury of the TMJ, (5) concurrent injury of the orbit/eye and TMJ, (6) concurrent injury of the orbit/eye and fracture of the mandible (excluding TMJ), and (7) concurrent injury of the orbit/eye, fracture of the mandible and injury of the TMJ. For the TMJ, the term injury was chosen instead of fracture because one case had a subluxation of the TMJ. Body injury was grouped as follows: (1) no injury (excluding the head), (2) thoracic injury, (3) orthopedic injury (limbs and spine), and (4) concurrent orthopedic and thoracic injuries. For all pairwise association the p statistic and the spearman rho coefficient were reported.

## Results

Twenty-five cats were included in the study. The median age was 21 months (range, 3–158 months). Twenty cats (80%) were 4 years of age or younger. The median body weight was 5 kg (range, 2.6–6.5 kg). Nine cats (36%) were female, of which one was intact, and 16 cats (64%) were male, of which three were intact. There were 23 domestic shorthair cats, one domestic longhair cat, and one Turkish Angora cat.

Causes of the PDE were falling from a height (*n* = 17), animal altercation (*n* = 2), and hit by car (*n* = 2). The cause was unknown in four cats, although it was suspected to be a fall from a height in one of them and hit by car in another (based on other concurrent injuries). All cats had other concurrent dental, oral, and maxillofacial injuries. Ten cats (40%) had orbital or ocular injury. Thirteen cats (52%) had injuries in other body parts (excluding the head) (Table 1). A full description of the lesions is beyond the scope of this study; concurrent injuries in high-rise syndrome and maxillofacial trauma have been described elsewhere (1–3).

**Table 1:** Cause of the acquired midline PDE and location of other injuries.

**Fall from a height**	**Animal altercation**	**Hit by car**	**Unknown cause**	**Other oral, facial and/or head injuries**	**Other body part injuries (excluding head)**
17/25	2/25	2/25	4/25	25/25	13/25
68%	8%	8%	16%	100%	52%

*PDE, Palate defect*.

The most common PDE extended from the level of the mesial aspect of the maxillary third premolar teeth (*n* = 8; 32%) or the incisor teeth (*n* = 7; 28%) caudally into the soft palate. Five cats (20%) had a PDE extending from the level of the mesial aspect of the maxillary fourth premolar teeth to the soft palate. The least common extension was from the level of the maxillary canine (*n* = 1; 4%) or the second premolar teeth (*n* = 2; 8%) to the soft palate and from the maxillary fourth premolar teeth to the THSP (*n* = 1; 4%). The medical record did not provide accurate description of the rostral extent of the PDE in one cat, but both the hard and soft palate (30-mm long) were affected (Table 2).

**Table 2:** Extent of the acquired midline PDE and treatment.

**Location of PDE**	**Frequency (%)**	**Surgical technique**	**Frequency (%)**
I-SP	7/25 (28%)	BPF BPF + IQS 104-204 UPF + IQS 104-204	2/7 (28.6%) 4/7 (57.1%) 1/7 (14.3%)
C-SP	1/25 (4%)	Direct apposition + IQS 103-203*	1/1 (100%)
PM2-SP	2/25 (8%)	Direct apposition UPF	½ (50%) ½ (50%)
PM3-SP	8/25 (32%)	BPF Direct apposition UPF BPF + IQS 104-204	4/8 (50%) 2/8 (25%) 1/8 (12.5%) 1/8 (12.5%)
PM4-SP	5/25 (20%)	Direct apposition BPF UPF	2/5 (40%) 2/5 (40%) 1/5 (20%)
PM4-THSP	1/25 (4%)	UPF	1/1 (100%)
HP-SP**	1/25 (4%)	Direct apposition	1/1 (100%)

PDE, Palate defect; I, Incisor teeth; C, Canine teeth; PM2, Maxillary second premolar teeth; PM3, Maxillary third premolar teeth; PM4, Maxillary fourth premolar teeth; HP, Hard palate; SP, Soft palate; THSP, Transition between hard and soft palate; BPF, Bilateral pedicle flaps; UPF, Unilateral pedicle flap; IQS, Interquadrant splinting. *IQS 103-203 was applied to stabilize the fracture of the incisive bones, but not the median palatine suture. For statistical purposes, this case was included in the direct apposition group. **Exact extent was not recorded in the medical records.

All cats had dental radiography, and seven also had head CT performed. The separation of the median palatine suture was diagnosed in all cats, but the identification of other fractures of the bones of the hard palate was attempted only in cases with CT. The CT imaging findings included bilateral maxillary fractures and fractures of the bones of the nasopharynx and orbit. The average number of functional units affected per cat was 11 (range, 9–14) (Table 3). The separation of the median palatine suture extended rostral to the soft tissue defect, and the widest area of the bone gap was present at the caudal aspect of the hard palate in six cats and at the level of the palatine fissures in one cat. The only cat (of the seven that had CT) with multiple fractures of bones of the hard palate had sustained a bite injury.

**Table 3:** Functional regions^*^ fractured in seven cats with acquired midline PDE that had computed tomography (CT).

**Functional region**	**Bilateral**	**Unilateral**	**Total (%)**
Median palatine suture	-	7/7	100
Nasal bone	3/7	1/7	57.1
Incisive bone, maxilla, palatine bone	7/7	-	100
Orbit	7/7	-	100
Nasopharynx	7/7	-	100
Mid-zygomatic arch	2/7	1/7	42.9
Mandibular fossa	-	4/7	57.1
Brain case	1/7	1/7	28.6
Mandibular symphysis/parasymphyseal	-	3/7	42.9
Mandibular ramus	-	2/7	28.6
Condylar process (head)	1/7	4/7	71.4
Condylar process (neck)	-	2/7	28.6

* *Anatomical functional regions adapted from Tundo et al. (2019). PDE, Palate defect*.

The treatment was performed within 5 days following the traumatic injury, preceded by general stabilization of the 25 cats. Twenty-two cats received perioperative antibiotic therapy (ampicillin, amoxicillin-clavulanic acid, clindamycin, clindamycin with amoxicillin-clavulanic acid, cefpodoxime or cefotaxime, and clindamycin with cefazolin). An esophagostomy feeding tube had been placed in six cats due to the extent of maxillofacial trauma, but not necessarily because of the presence of the PDE.

**Figure 1:**
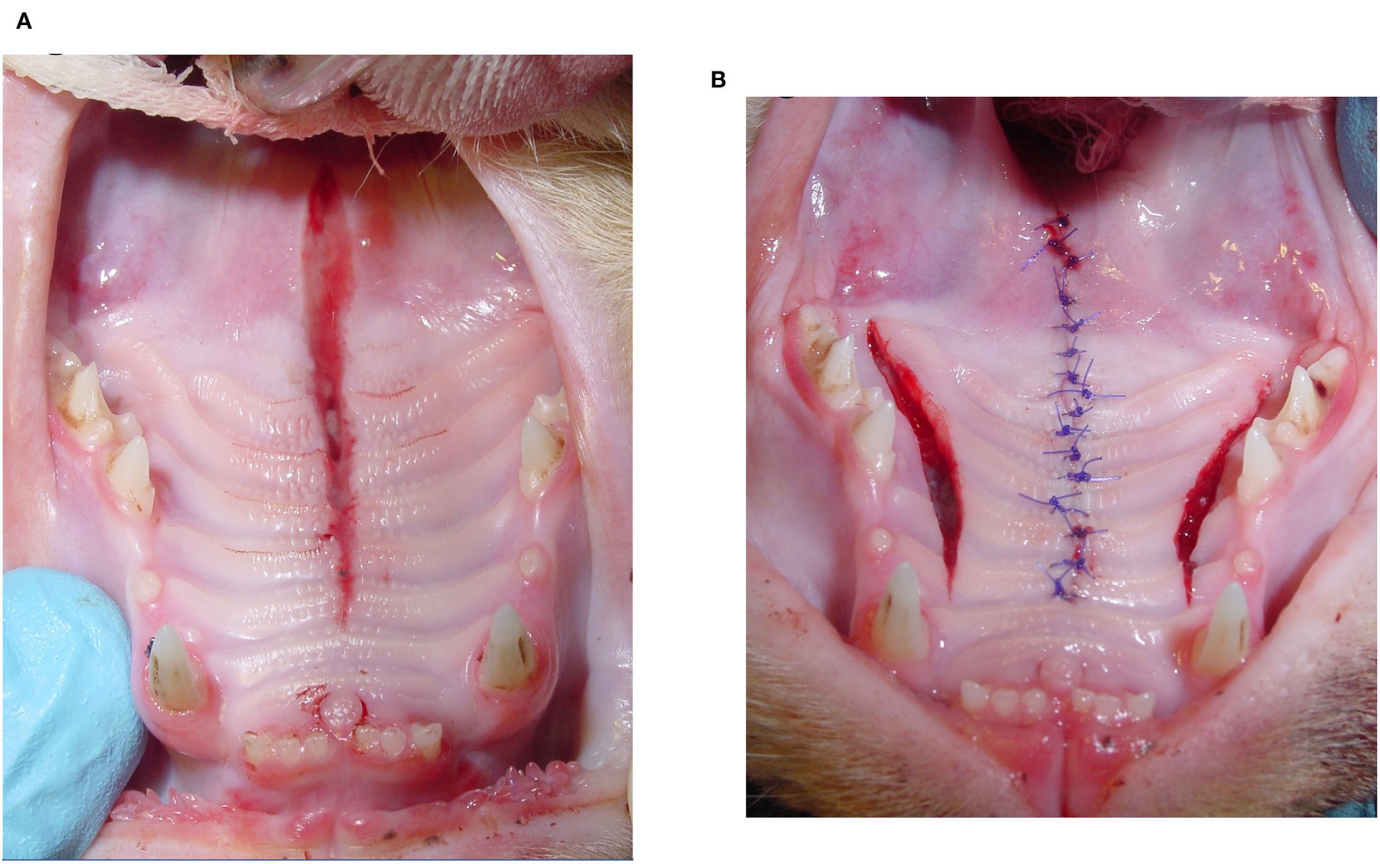
Bilateral pedicle flap technique with lateral releasing incisions. **(A)** Acquired midline palate defect (PDE) extending from the level of the mesial aspect of the maxillary second premolar teeth to the rostral aspect of the soft palate. Increased interdental space between the right and left maxillary first incisor teeth is caused by separation of the median palatine suture. **(B)** Bilateral releasing incisions were made to elevate the right and left pedicle flaps. The PDE was sutured closed in a simple interrupted pattern.

**Figure 2:**
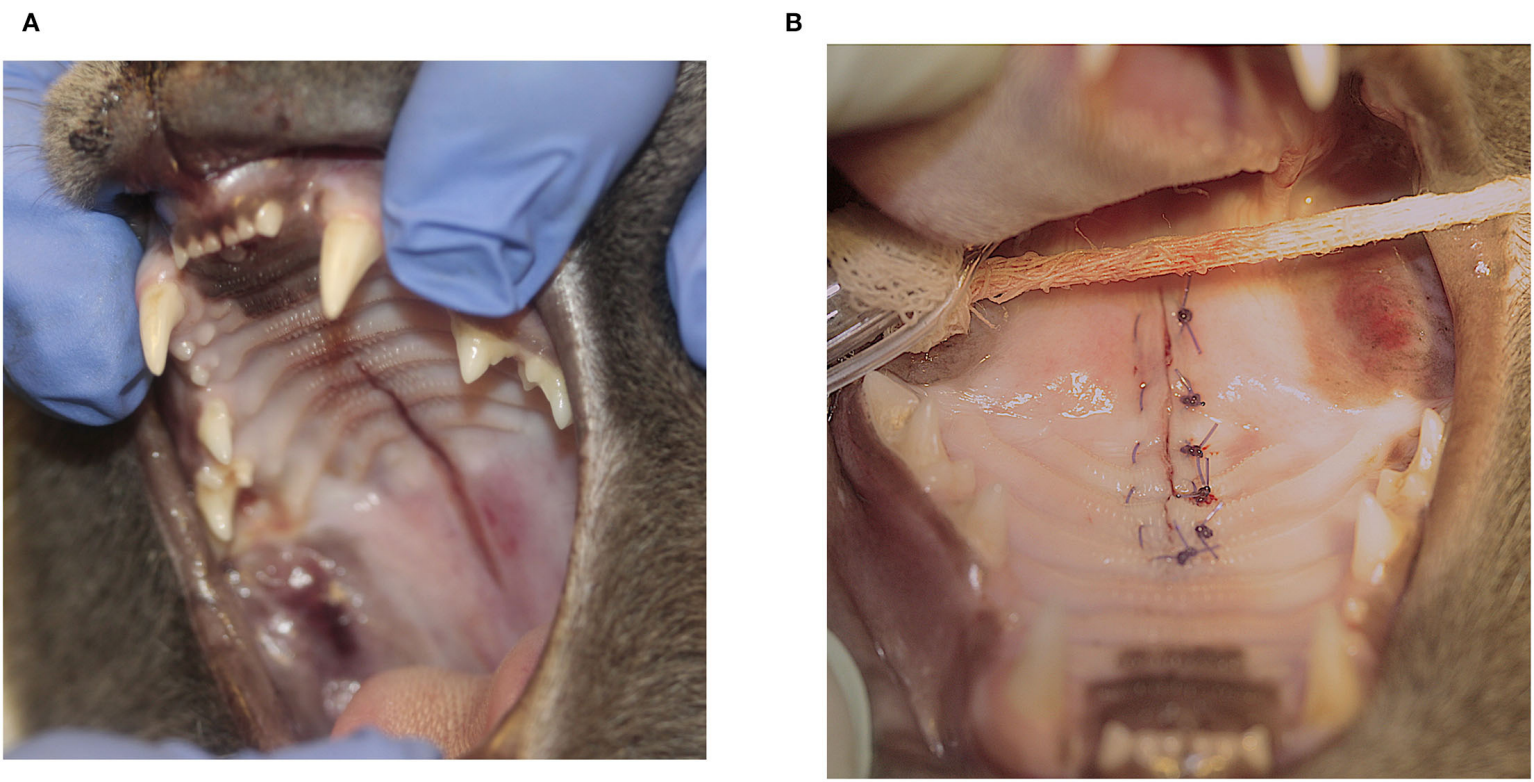
Direct apposition of the mucoperiosteal edges without releasing incisions. **(A)** Acquired midline palate defect (PDE) extending from the level of the mesial aspect of the maxillary third premolar teeth to the rostral aspect of the soft palate. **(B)** Direct apposition of the mucoperiosteal tissue edges in a horizontal mattress suture pattern.

**Figure 3:**
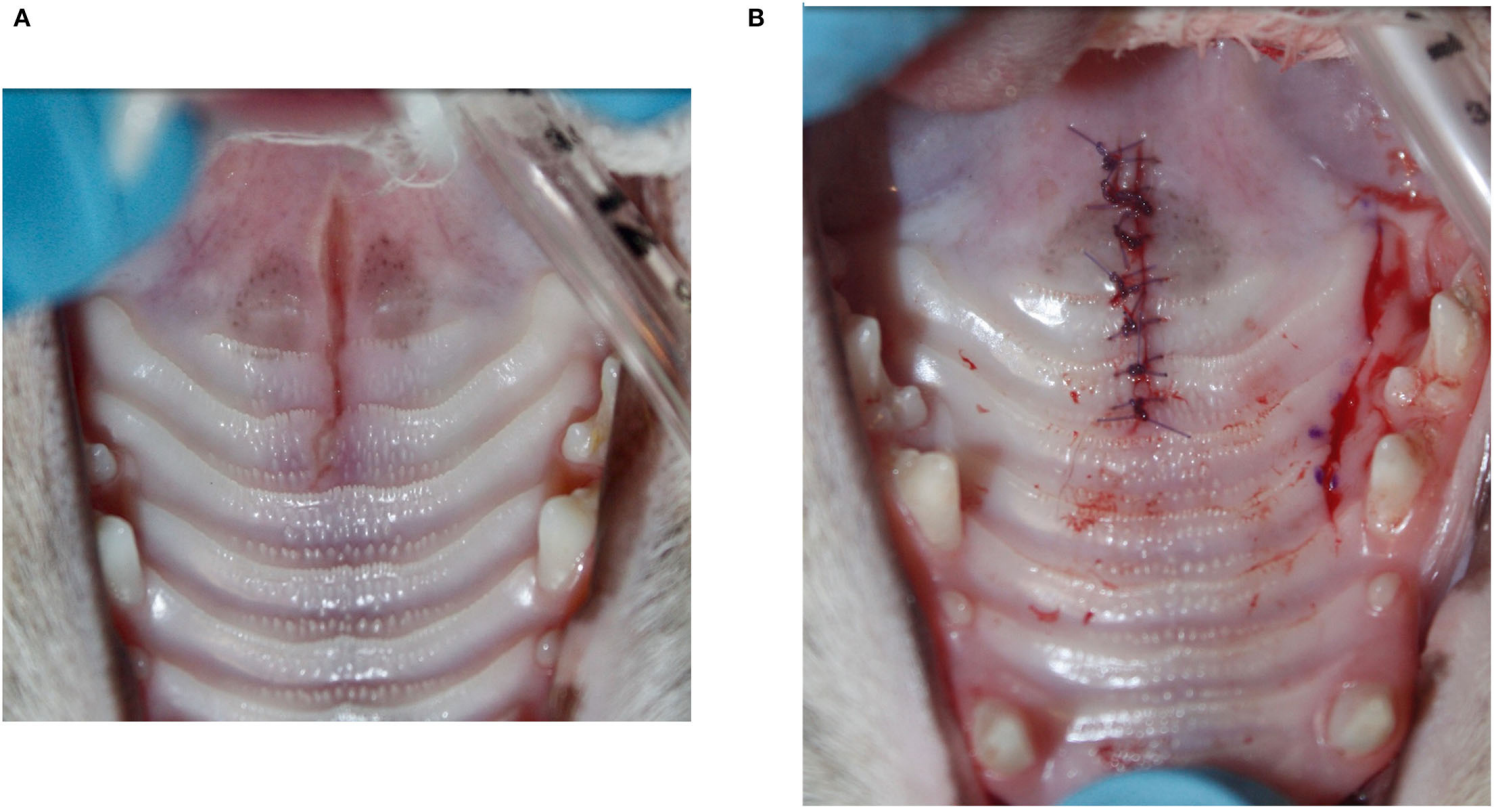
Unilateral pedicle flap technique with lateral releasing incision. **(A)** Narrow acquired midline palate defect (PDE) from the level of the maxillary fourth premolar teeth to the rostral aspect of the soft palate. The widest area is located at the transition of the hard and soft palate. **(B)** The unilateral releasing incision was made about 2-mm palatal to the teeth and did not extend into the soft palate. After elevation of the mucoperiosteal flap between the defect and the lateral incision, the PDE was sutured closed in a simple interrupted pattern.

**Figure 4:**
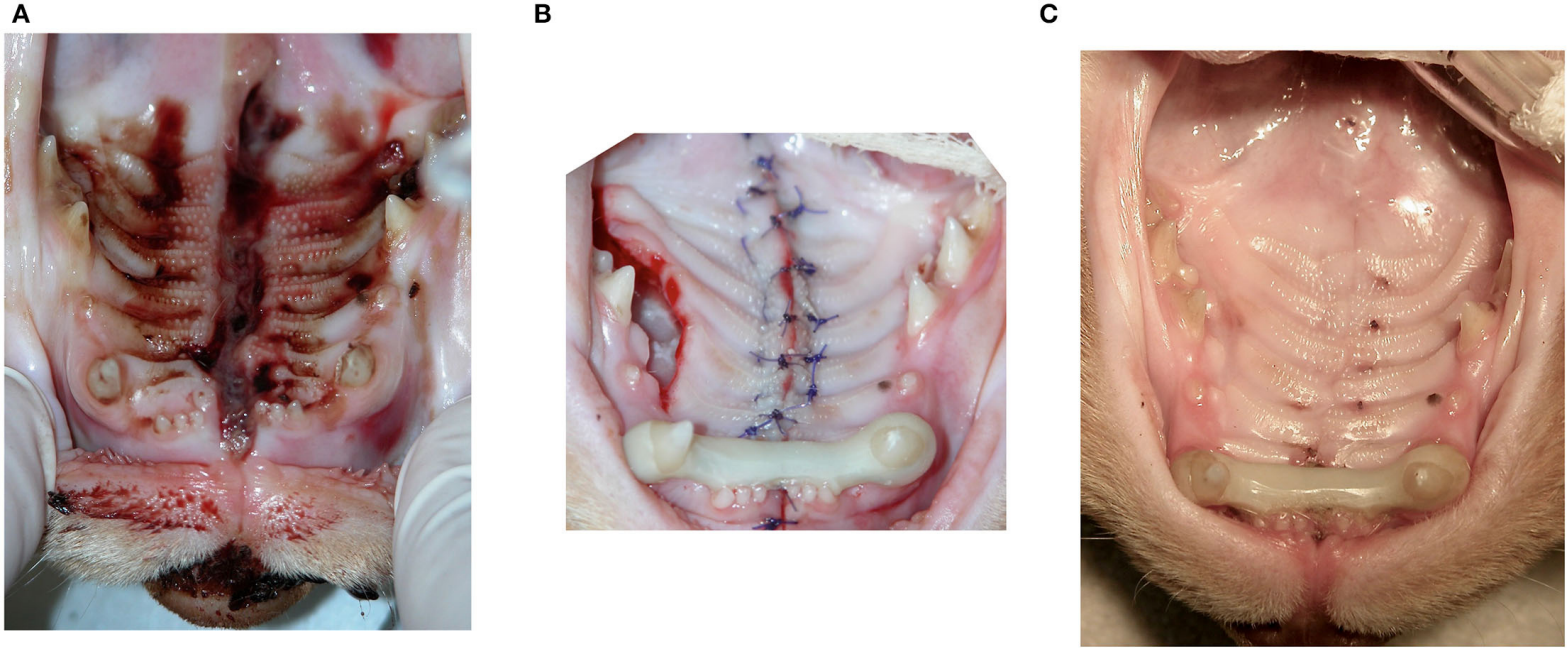
Unilateral pedicle flap technique with lateral releasing incision and the interquadrant splinting between the right and left maxillary canine teeth. **(A)** Wide acquired midline palate defect (PDE) extending from the level of the incisor teeth to the rostral aspect of the soft palate. **(B)** Following the closure of the mucoperiosteal tissue edges in one layer (alternating horizontal mattress and simple interrupted pattern), a splint between the right and left maxillary canines was placed to reduce and stabilize the median palatine suture separation. **(C)** Five-week postoperative image before removal of the splint. The PDE is healed, and the denuded bone in the area of the lateral releasing incision is completely epithelialized.

The most common techniques used to repair the PDE were medial mucoperiosteal apposition with bilateral pedicle flaps and lateral releasing incisions (*n* = 8; 32%) (Figure 1), direct medial mucoperiosteal apposition without lateral releasing incisions (*n* = 7; 28%) (Figure 2), medial mucoperiosteal apposition with bilateral pedicle flaps and lateral releasing incisions, and interquadrant splinting between maxillary canine teeth (*n* = 5; 20%), and medial mucoperiosteal apposition with a unilateral pedicle flap and lateral releasing incision (*n* = 4; 16%) (Figure 3). The PDE in one cat was repaired with one unilateral pedicle flap and lateral releasing incision and interquadrant splinting (*n* = 1; 4%) (Figure 4; Table 2). The maxillary canine teeth with a complicated crown fracture (*n* = 3) were included in the interquadrant splinting if they had sufficient coronal length. The vital pulp therapy at the time of PDE repair and subsequent root canal treatment upon splint removal was performed in one tooth. The other two teeth had no initial treatment and were extracted at the time of splint removal. The laceration in the soft palate was closed by direct apposition of the soft tissue edges after a careful undermining of the oral mucosa to obtain a tension-free closure in all cats.

A one-layer closure was used for the PDE repair in 15 cats (60%) (12 with a simple interrupted pattern and three with an alternating horizontal mattress and simple interrupted pattern). The PDE of seven cats (28%) was repaired with a two-layer closure; a horizontal mattress pattern for the connective tissue of the hard palate and the nasal mucosal–muscular (soft palate) layer and a simple interrupted pattern for the oral mucosa of the soft palate. The PDE of one cat (4%) was repaired with one layer of sutures in the hard palate and three layers of sutures in the soft palate (nasal mucosa, muscle tissue, and oral mucosa). There was insufficient information in the medical records about the suture patterns used in two cats (8%). The PDE was closed with 4–0 or 5–0 poliglecaprone 25 in 19 cats (76%) or polydioxanone in four cats (16%). There was an insufficient information in the medical records about the type or size of the absorbable monofilament suture material used in two cats (8%).

The patients were discharged with instructions to be fed a soft diet for 2 weeks (*n* = 19; 76%) or provided nutrition through the esophagostomy feeding tube (*n* = 6; 24%). The pain medication was tailored to each cat depending on the additional injuries, and all cats wore an E-collar to prevent grooming for at least 2 weeks.

The information about healing rechecks was available for 19 of the 25 cats (76%). A good outcome was observed in all cases that had follow-up visits (*n* = 19; 100%) with no complications associated with the healing of the PDE. None of the patients was diagnosed with an oronasal fistula at any of the recheck examinations. Malocclusion was noted in two cats (10.5%). One of them had palatoversion of the maxillary premolar/molar teeth due to displacement of multiple bone fragments of the midface causing alveolar mucosal trauma at the ipsilateral mandible. Extraction of the malocclusion maxillary teeth resolved the soft tissue trauma. The malocclusion in the second cat was mild, and further treatment was not necessary.

All PDE healed by primary healing. The areas of denuded bone resulting from the lateral releasing incisions healed by the second intention without complications. The food debris accumulation over granulation tissue at these lateral releasing incisions was visible up to 13 days after surgery in two cats which was managed by rinsing the mouth with water or antiseptics after feeding. One out of the 19 cats with recheck information was euthanized 7 days after the PDE repair due to sepsis secondary to a spinal cord injury with paralysis and bladder dysfunction. The PDE already had healed at that time. The mean time for visual evidence of healing of the PDE was 20 ± 14 days (range, 6–59 days). A bed of granulation tissue at the lateral releasing incisions was visible in some cats as early as 10 days after surgery.

The interquadrant splinting showed association (*p* < 0.05) with the following seven variables: Time to repair, presence of symphyseal separation, head injury, body injury, orthopedic injury, thoracic injury, and extent of the PDE. For the outcome of complications, only the variables TMJ injury, use of a feeding tube, and having a CT performed were significantly associated with the outcome. Having a CT performed showed association with the presence of symphyseal separation, orbital/ocular, TMJ and head injury, use of a feeding tube, and time to repair (Table 4).

**Table 4:** Correlation between independent variables (Spearman rank test, *p* < 0.05).

**Variable 1**	**Variable 2**	***P***	Rho^*^
Interquadrant splint	Time to repair (days)	0.0140	0.6925
	Symphyseal separation	0.001	0.6925
	Head injury	0.0194	0.4663
	Orthopedic injury	0.0359	−0.4215
	Body injury	0.0051	−0.5427
	Thoracic injury	0.0211	−0.4588
	Extent of the PDE	0.0022	−0.5833
Complications	TMJ injury	0.0071	0.5247
	Feeding tube	0.0071	0.4839
	CT	0.0170	0.4729
CT	Symphyseal separation	0.0447	0.4048
	Orbital/ocular injury	0.0000	0.7638
	Head injury	0.0000	0.7895
	TMJ injury	0.0001	0.6925
	Complications	0.0170	0.4729
	Feeding tube	0.0142	0.4839
	Time to repair (days)	0.0046	0.5471

* *Rho is the Spearman correlation statistic. It varies from −1 to 1 where, 1 indicates high correlation and −1 high inverse correlation. Value of 0 means no correlation. PDE, Palate defect; CT, Computed tomography; TMJ, Temporomandibular joint*.

## Discussion

The results of this retrospective study showed that acquired midline PDE occur most frequently after falling from a height with or without concurrent lesions associated with high-rise syndrome (i.e., thoracic and limb injuries). Repairing the PDE soon after stabilization of the patient has a good outcome with a low frequency of complications. The data suggest that the treatment chosen for each case was not only dependent on the extent of the PDE but also on the stability and separation of the bone fragments of the palate and the ability to achieve a tension-free closure. The defects extending rostrally in the hard palate benefit from stabilization of the midface with an interquadrant splint.

An oral examination is sufficient to diagnose the presence of a PDE. If the separation of the tissue edges and the oronasal communication are not readily evident, a PDE can be verified by gentle evaluation of the wound with a periodontal probe under sedation or general anesthesia. An abnormal interdental space between the maxillary first incisors and instability of the midface can be easily evaluated by inspection and palpation. A complete dental and maxillofacial assessment must be performed to establish a plan of treatment for the other maxillofacial injuries. In the previous studies, ~70% of dogs and cats with maxillofacial trauma also sustained dental trauma (1, 2, 9). Some injuries may not be as evident as a jaw fracture, but they should be considered a potential source of pain and thus be treated accordingly.

Many of the cats of this study sustained pulmonary contusion or other lesions that involved intensive care, additional diagnostic tests, multiple surgical procedures, or more involved management. A head CT might not have been prioritized due to reasonably good health status of the patient, minimal clinical signs related to the fractures of the midface (i.e., patient able to open and close the mouth, no evidence of ocular problems, absence of neurologic signs, and minimal facial asymmetry) and additional cost. The recent imaging studies have shown that midface fractures tend to occur simultaneously with a median of eight regions of the face affected at the same time (2, 10). The separation of the median palatine suture occurs frequently with fractures of the orbit, nasopharynx, and nasal bones (2). In the presence of maxillofacial/head trauma, a CT is recommended for full assessment of any fractures (2, 4, 10). The cats that had CT performed in this study presented with more severe clinical findings which explains the association found between CT imaging and the presence of more severe head injury, orbital or ocular injury, symphyseal separation, and placement of a feeding tube. It may also reflect the evolution of the imaging recommendations at our institution.

A conservative treatment with antibiotic therapy and feeding a soft diet for at least a month was recommended in a study with 22 high-rise syndrome cats presenting with an acquired midline PDE, stating that all cases healed by second intention without signs of oronasal fistula. However, the authors also mentioned that they had previously seen chronic oronasal fistulae secondary to a PDE that were not surgically repaired (7). The epithelialization and contraction of the wound when the palatal mucoperiosteal edges heal will lead to a more scarred and less elastic tissue available for the repair and a slightly wider oronasal fistula, making the delayed PDE repair more challenging (1, 11). Some tissue removal at the epithelialized edge of a chronic PDE to freshen up the flap edges will also increase the width of the final defect prior to the closure. Furthermore, the palatine fissures and transition of the hard and soft palate (THSP) are known to be areas with higher risk of oronasal fistula formation after the PDE repair (5, 12). These areas also correspond to the wider gaping between the separated bones in the midline as was seen in the cats assessed by CT in this study. Therefore, a special attention should be given to these locations during surgery to avoid tension at the suture lines.

A study conducted in rats showed that when the palatal mucosa incision is left to heal by second intention, an oronasal fistula would result in up to 85% of the animals when the distance between the bony edges was 2 mm and in up to 33% of the animals when the bony defect was as small as 1 mm. During healing by second intention, the nasal mucosa may grow into the space of the bony defect, eventually contacting and fusing with the palatal mucosa and forming an oronasal fistula (13). That study, while done in a different species, underscores the importance of prompt surgical intervention, even in the case of a narrow PDE.

The surgical methods for the PDE repair after wound debridement include direct medial mucoperiostal apposition of the soft tissue edges, medial mucoperiosteal apposition with one or two pedicle flaps (and one or two lateral releasing incisions) with or without interquadrant splinting or a tension band with a pin and wire (1, 3, 5, 8).

The surgical technique should adhere to the following general principles of palate surgery: (1) Avoid tension on the suture line by creating flaps that are larger than the defect; (2) Maintain the vascular supply of the flaps by preserving the major palatine artery, which in cats arises from the major palatine foramen under the palatal mucosa at the level of the mesial aspect of the maxillary fourth premolar tooth halfway between the dental arch and median plane; (3) Suture only fresh edges of soft tissue together; (4) Avoid electrosurgery or cauterization when controlling hemostasis; and (5) handle the tissues gently to minimize trauma. An attempt should be made to keep the suture lines over connective tissue and bone instead of over a void (14).

Successful outcome of a PDE surgery can be expected if the trauma was acute, the defect in the midline, the tissue edges nicely vascularized, the major palatine arteries intact, and surgical repair performed without delay. The techniques used in this retrospective study were based on the judgment of the primary clinician with the intention of avoiding tension in the suture line. There was no significant difference in the outcome (healing *vs*. no healing) between the use of a simpler technique (e.g., direct medial mucoperiosteal apposition) vs. a more complicated one (e.g., pedicle flaps and releasing incisions with or without interquadrant splinting) because each technique was carefully selected on a case-by-case basis, trying to follow the general principles of palate surgery. The separation of the median palatine suture with more severe displacement and instability likely warranted a more aggressive treatment protocol (bilateral pedicle flaps with releasing incisions and interquadrant splinting).

The only surgical principle that could not be followed was to keep the suture line over the connective tissue or bone. This did not seem to be a problem in the group of cats evaluated in this study. The adherence to all the other principles of palate surgery and good vascularization of the acutely injured palatal mucoperiosteum contributed to the success of the PDE repair. However, the results of this study (100% success in the cases with follow-up visits) may not reflect the outcome of all cases with acquired midline PDE. Comminuted fractures of the bones of the hard palate may cause wider bone gaps and decreased bone support for the soft tissues used for the closure, increasing the risk of dehiscence or development of secondary lateral PDE if lateral releasing incisions are used for a tension-free closure in the midline. If loose bone fragments are retrieved from the PDE, the addition of a barrier material (e.g., auricular cartilage, fascia lata, flexible bone membrane, porcine submucosa membrane) between the remaining bone and palatal mucoperiosteum could facilitate successful outcome. Therefore, full characterization with oral examination and head CT and careful surgical planning of each case should be made before attempting the PDE repair with the techniques described in this study.

On the statistical analysis, the only treatment-related variable that was associated with the extent of the PDE was interquadrant splinting between the maxillary canine teeth. Interquadrant splinting may reduce and stabilize the separation of the median palatine suture in the more rostral part of the hard palate at the level of the incisor, canine, and second premolar teeth. Therefore, based on the cases evaluated in this study, PDE extending into the rostral half of the hard palate may benefit from interquadrant splinting. However, splinting may not be necessary if the PDE is located in the caudal half of the hard palate (between the palatine bones) unless the separation of the median palatine suture extends farther rostrally. In addition, the number of lateral releasing incisions is subjectively decided by the primary clinician based on PDE width and the ability to achieve a tension-free closure. There was insufficient information in the medical records to determine the cut-off value of PDE width.

The only complication observed was malocclusion, which was associated with concurrent injury of the TMJ. The TMJ injury was treated conservatively. Even a mild displacement of bone fragments can cause malposition of the canine or premolar/molar teeth. This, in addition to the malalignment of the injured midface, could result in malocclusion and difficulty to fully close the mouth. The prevalence of malocclusion may be underestimated if some teeth were extracted at the time of the PDE repair or interquadrant splint removal, eliminating the possible tooth-to-tooth contact or tooth-to-soft tissue contact. Malocclusions secondary to other maxillary and mandibular fractures may be more common, but if they did not cause any obvious clinical signs, they may not have been recorded in the medical record or observed as such by the clinician. The cats sustaining a fall from a height tend to hit the ground with their limbs first, followed by the thorax, and then the head. Several mechanisms have been suggested to explain the resulting maxillofacial injuries (1). The impact causes a fracture with separation in the midline of the hard palate. Because the upper dental arch is wider than the lower dental arch (anisognathism), an increase in the distance between the right and left maxillary premolar/molar teeth may not cause any obvious or consequential traumatic occlusal contact.

It is possible that more cats in this study could have had complications that were simply not identified because of the lack of medium to long-term follow-up. The presence of complications also was significantly associated with CT performed and feeding tube placed. As suggested before, this may reflect the decision made by the primary clinician to better manage cats with worse injuries diagnosed. The TMJ injury is frequently treated with maxillomandibular fixation, muzzling, sutures through labial buttons, or elastic chains (5, 6, 15). Placement of a feeding tube is likely used in these patients. It remains unknown whether the use of CT would have changed the treatment plan if it had been performed in all cases.

The average healing time of the PDE in this study was 20 days. However, this number does not reflect the actual time needed for palatal mucoperiosteum to heal, as the healing time assessed was based on recheck examinations at follow-up visits. The cats were typically evaluated by means of an awake oral examination 2 weeks post-surgery or in 4–8 weeks for the removal of the interquadrant splint and further dental treatment. Healing of the oral mucosa has been extensively studied in cleft palate models in dogs, where the midline defect healed quickly within 1 week by first intention after accurate apposition of the soft tissue edges, while the lateral incisions healed in ~ 3–5 weeks by second intention with clot formation followed by granulation tissue, epithelialization, and scar formation (16–18). Mucoperiosteal defects of the hard palate of 1-cm diameter had almost completely healed by 7 days, but the center takes a few more days of healing (19). In this study, a mild inflammation was visible around the suture material when the follow-up was performed between 1 and 2 weeks as part of the normal healing process of the palatal mucoperiosteum or due to irritation and accumulation of debris around the sutures.

In acute midline PDE described in this study, there is usually minimal soft tissue loss; thus, the amount of denuded bone, if one or two pedicle flaps are made, is minimal compared to the studies on cleft palate models. Therefore, healing (epithelialization and lack of inflammation) of the lateral incisions typically occurs within 2 weeks which is consistent with our clinical observations. In experimental studies in dogs, incisions made in the palatal mucosa without elevating the mucosa from the periosteum or without causing bone loss healed by second intention quickly in a similar fashion to first intention, leaving a small scar of 0.5 mm wide (17, 18). The epithelialization over denuded bone progressed from the periphery toward the center of the defect, advancing faster from the lateral aspect of the defect than the medial aspect (14). The epithelium was thinner than in healthy palatal mucosa, and the firmly attached connective tissue lacked elastic fibers in the early weeks of healing (16, 17, 20). The soft palate healed quickly. The dissection and reconstruction of the muscular layer of the soft palate in cats did not cause permanent structural damage to the muscular fibers or fibrosis. Furthermore, the recovery of the normal structure of the muscles of the soft palate was reported to occur within 3 months after the surgical procedure (21).

This study has some limitations. The medical records may not have contained all the details we would have liked to include in the study, and the follow-up visits were not standardized. The surgical technique used to repair the PDE was biased due to clinician's experience and preference for one technique or another. Computed tomography imaging was not used in all patients; therefore, some injuries of the midface or the TMJ may have been missed. Small oronasal fistulae may not have been diagnosed because healing by means of probing the surgical site was not assessed under general anesthesia in every patient.

This retrospective study showed that treatment of an acquired midline PDE with the techniques reported here was highly successful. Cats with a PDE extending rostrally to the level of the maxillary third premolar tooth may benefit from the addition of an interquadrant splint constructed between the maxillary canines. The maximum width at which the PDE can be left to heal by second intention and the number of releasing incisions required based on PDE width still needs to be determined. Evaluation of complete medical records, with accurate measurements of defects in the mucoperiosteum and bone, of cats with and without primary closure of the PDE may elucidate this information.

## Data Availability Statement

The raw data supporting the conclusions of this article will be made available by the authors, without undue reservation.

## Ethics Statement

Ethical review and approval was not required for the animal study because the retrospective study used information retrieved from medical records. Written informed consent for participation was not obtained from the owners because it was not deemed necessary due to the retrospective nature of the study.

## Author Contributions

AC-G: study concept and design, acquisition of data, analysis and interpretation of data, drafting of manuscript, and revision of the manuscript. DS: analysis and interpretation of data and statistical analysis. AR: study concept and design, acquisition of data, and critical revision of the manuscript for important intellectual content. All authors contributed to the article and approved the submitted version.

## Conflict of Interest

The authors declare that the research was conducted in the absence of any commercial or financial relationships that could be construed as a potential conflict of interest.

## Publisher's Note

All claims expressed in this article are solely those of the authors and do not necessarily represent those of their affiliated organizations, or those of the publisher, the editors and the reviewers. Any product that may be evaluated in this article, or claim that may be made by its manufacturer, is not guaranteed or endorsed by the publisher.
